# Supplementary material for: Beyond clustering: mean-field dynamics on networks with arbitrary subgraph composition
Source: J Math Biol. 2015 Apr 17;72:255–81. doi: 10.1007/s00285-015-0884-1 (PMC4698307; doi:10.1007/s00285-015-0884-1)
Supplement: Supplementary file 1 — Supplementary material 1 (zip 20 KB) [file 285_2015_884_MOESM1_ESM.zip › PGF_equation_generator.html]

PGF\_equation\_generator 

## Contents

- SIR-ODE generation for hyperstub configuration model networks
- Example usage
- Initialisation
- PGF generation
- Code generation
- State transition matrix generation

## SIR-ODE generation for hyperstub configuration model networks

```
function PGF_equation_generator(lambda, varargin)
```

```
% ------------------------------------------------------------------------
%                    http://arxiv.org/abs/1405.6234
% ------------------------------------------------------------------------
% The following code will generate generating function bassed,
% deterministic ODEs and corresponding solutions. The solutions include
% population level averages for the three respective compartments that may
% be compared to averages taken from simulation. Written by Martin Ritchie,
% University of Sussex, 2014.
% ------------------------------ output ----------------------------------
% lambda:   a vector containing the desired expected subgraph count per
%           node, lambda(i) corresponds to the ith varargin,
% varargin: Input is a string, where each string is the name assoicated the
%           desired subgraph. Separate different subgraphs names with
%           commas. Input subgraphs in the same order as thier given
%           expectations in lambda (example subgraphs are given in
%           subgraphs.mat).
% ------------------------- Generated files ------------------------------
% x_alpha.m (one per subgraph type),
% x_equations.m (one per subgraph type),
% PGF_jacobian.m (one for the whole system)
% PGF_hessian.m (one for the whole system)
% func.m (one for the whole system)
%
% # dependencies: combinator.m, inf_neighbors.m, subgraphs.mat
% and MATLAB's symbolic toolbox.
```

## Example usage

1. The following will generate and solve ODEs for a Poisson random network with parameter, lambda = 4:

PGF\_equation\_generator(4, 'C2'); [S, I, R, T] = episolve();

Where 'C2' denotes a complete subgraph of two nodes, i.e. a line, that will be loaded from subgraphs.mat. Load subgraphs.mat to see what subgraphs are available or create your own.

1. The following will generate and solve ODEs for a network where lines and triangles are Poisson distributed with parameters, lambda = [2 2] respectively:

PGF\_equation\_generator([2 2], 'C2', 'C3'); [S, I, R, T] = episolve(); Note that the epidemic parameters are controlled from within this function.

## Initialisation

```
global eps tau gamma Tend PGF_Jacobian_1 sg node_positions alpha
load('subgraphs',varargin{:});
% Note that the epidemic parameters are controlled from within this
% function.
% eps: fraction of inital infected.
eps = 1/10000;
%
Tend = 15;
% tau: per link rate of infection.
tau = 1;
% gamma: rate of recovery.
gamma  = 1;
% node_positions: number of positions in the system.
node_positions = 0;
% sg: a cell array where each entry contains the adjacency matrix of the
%     subgraphs specified by varargin.
for i = 1:length(varargin)
    sg{i} = eval(varargin{i});
    node_positions = node_positions + length(sg{i});
end
```

## PGF generation

The following utilises MATLAB's symbolic toolbox. It symbolically generates and compute the Jacobian and Hessian of the PGF that generates the networks subgraph degree distribution. In this implementation each subgraph is Poisson distributed. ------------------------------------------------------------------------

```
%------------------------ Standard Poisson PGF ----------------------------

% The following generates the PGF.
X = sym('X', [1 node_positions]);
PGF = 1;
for i = 1:length(sg)
    m(i) = length(sg{i});
    PGF = PGF*exp(lambda(i)*m(i)^(-1) * (sum(X(1:m(i)))-m(i)));
    X(1:m(i)) = [];
end
X = sym('X', [1 node_positions]);

% It is inefficient to keep it in symbolic form. The following converts
% the symbolic forms of the Jacobian and Hessian of the PGF into MATLAB
% functions.

PGF_Jacobians = jacobian(PGF,X);
PGF_Hessians = hessian(PGF,X);
% Convert the symbolic Jacobian and Hessian to .m MATLAB functions.
matlabFunction(PGF_Hessians, 'file', 'PGF_Hessian','vars', X);
matlabFunction(PGF_Jacobians, 'file', 'PGF_Jacobian','vars', X);
clear PGF_Jacobians PGF_Hessians

% % state_count(i):    The number of states associated with subraph i.
% % subgraph_index(i): The first position index associated with each subgraph.
state_count = zeros(length(varargin),1);
subgraph_index = zeros(length(varargin),1);
for i = 1:length(varargin)
    if i ==1
        subgraph_index(i) = 1;
    else
        subgraph_index(i) = subgraph_index(i-1) + length(sg{i-1});
    end
    state_count(i) = 3^length(sg{i});
end
% The total number of ODEs:
system_size = (sum(state_count) + node_positions + 2);
% PGF_Jacobian_1: Jacobian of the PGF evaluated at 1.
in = num2cell(ones(1,node_positions));
PGF_Jacobian_1 = PGF_Jacobian(in{:});
```

```
Error using mupadmex
Error in MuPAD command: The number of columns does not match. [(Dom::Matrix(Dom::ExpressionField()))::mkSparse]

Error in sym/jacobian (line 34)
Jsym = mupadmex('symobj::jacobian',F.s,x.s);

Error in PGF_equation_generator (line 91)
PGF_Jacobians = jacobian(PGF,X);
```

## Code generation

The following generates three different m-files: # x\_alpha.m: a function file that returns initial conditions for the subgraph x, # x\_equations: a function file that returns state equations for the subgraph x, # func.m: the function that is passed to ODE45 for integration. alpha: is a vector of initial conditions that is eventually passed to ode 45. It needs to be initialised outside of the following loop, within which the remaining initial conditions are generated and appended to alpha.

```
alpha = zeros(1,(node_positions));
% Survivor function (theta) initial conditions:
for i = 1:node_positions
    if PGF_Jacobian_1(i)~=0
        alpha(i) = 1;
    end
end

% ii cycles through each subgraph generating x_equations.m followed by
% x_alpha.m
for ii = 1:length(varargin)
    % states: a matrix containing all possible states of g.
    states = combinator(3,length(sg{ii}),'p','r')-2;
    % trans_matrix: creates the state transition matrix for g.
    transition_matrix = trans_matrix(sg{ii},subgraph_index(ii),states);

    % the following creates a matlab function file that corresponds to
    % the state equations for subgraph g.
    flux_name = sprintf('%s_equations.m',varargin{ii});
    fid = fopen(flux_name,'w');
    ltm = length(transition_matrix);
    fprintf(fid,'function [dy] = %s_equations(y) \n \n global tau gamma Delta M \n D = Delta; \n \n ',varargin{ii});
    for i = 1:ltm
        for k = 1:length(sg{ii})
            switch states(i,k)
                case -1
                    s(k) = 'S';
                case 0
                    s(k) = 'I';
                case 1
                    s(k) = 'R';
            end
        end
        fprintf(fid, '  %% %s \n ', s);
        fprintf(fid,'dy(%d) = ', i);
        for j = 1:ltm
            if ~isempty(transition_matrix{i,j})
                if transition_matrix{i,j}~=0
                    fprintf(fid,' - y(%d)*(%s)', [i transition_matrix{i,j}]);
                end
            end
            if ~isempty(transition_matrix{j,i})
                if transition_matrix{j,i}~=0
                    fprintf(fid,' + y(%d)*(%s)', [j transition_matrix{j,i}]);
                end
            end

        end
        fprintf(fid,';\n \n');
    end
    fprintf(fid,'end');
    fclose(fid);

    % Initial conditions: the following generates x_alpha.m, a matlab
    % function file that returns a vector of initial conditions for subgraph
    % x.

    alpha_name = sprintf('%s_alpha.m',varargin{ii});
    fid = fopen(alpha_name,'w');
    ltm = length(transition_matrix);
    fprintf(fid,'function [initial] = %s_alpha \n \n global eps PGF_Jacobian_1 \n',varargin{ii});
    fprintf(fid,'initial = zeros(1,%d); \n', ltm);
    fprintf(fid,'if PGF_Jacobian_1(%d)==0 \n', subgraph_index(ii));
    fprintf(fid,'\t return \n');
    fprintf(fid, 'else \n');
    for i = 1:ltm
        if isempty(find(states(i,:)==1))
            s_count = 0;
            i_count = 0;
            r_count = 0;
            for k = 1:length(sg{ii})
                switch states(i,k)
                    case -1
                        s_count = s_count + 1;
                    case 0
                        i_count = i_count + 1;
                    case 1
                        r_count = r_count + 1;
                end
            end

            if s_count == length(sg{ii})
                fprintf(fid,'initial(%d) = (1-eps)*PGF_Jacobian_1(%d); \n', [i subgraph_index(ii)]);
            elseif i_count >= 2
                fprintf(fid,'initial(%d) = 0; \n', i);
            elseif i_count == 1
                fprintf(fid,'initial(%d) = eps*PGF_Jacobian_1(%d); \n', [i subgraph_index(ii)]);
            else
                fprintf(fid,'initial(%d) =0; \n', [i]);
            end

        end
    end
    fprintf(fid, 'end \n');
    fprintf(fid,'end');
    fclose(fid);
    innit_name{ii} = sprintf('%s_alpha',varargin{ii});
    alpha(end+1:end + 3^length(sg{ii})) = feval(innit_name{ii});
end
% I_0
alpha(end+1) = eps;
% R_0
alpha(end+1) = 0;


% Generation of func.m: func.m is the ODE function that is passed to ODE45
% along with the vector of initial conditions, 'alpha'.
fid=fopen('func.m','w');
fprintf(fid,'function dy = func(~,y) \n \n');
fprintf(fid,'global tau gamma Delta M PGF_Jacobian_1 \n');
fprintf(fid,'T  = zeros(1,%d); \n ',node_positions);
fprintf(fid,'dy = zeros(%d,1); \n \n',system_size);

for i = 1:node_positions
    if i==1
        fprintf(fid, 'PGF_Jacobian_theta  = PGF_Jacobian(y(%d),', i);
    elseif i==node_positions
        fprintf(fid, 'y(%d)); \n', i);
    else
        fprintf(fid, 'y(%d),', i);
    end
end
for i = 1:node_positions
    if i==1
        fprintf(fid, 'PGF_Hessian_theta  = PGF_Hessian(y(%d),', i);
    elseif i==node_positions
        fprintf(fid, 'y(%d)); \n', i);
    else
        fprintf(fid, 'y(%d),', i);
    end
end
% For loop that generates susceptible excess degree matrix, delta:
fprintf(fid, 'for i = 1:%d \n', node_positions);
fprintf(fid, ' \t M(i) = y(i)*PGF_Jacobian_theta(i); \n');
fprintf(fid, '\t for  j = 1:%d \n',node_positions);
fprintf(fid, '\t\t if PGF_Jacobian_theta(i)==0 \n');
fprintf(fid, '\t\t\t delta(i,j)=0; \n');
fprintf(fid, '\t\t else \n');
fprintf(fid, '\t\t\t delta(i,j) = y(j)*PGF_Hessian_theta(i,j)/PGF_Jacobian_theta(i); \n');
fprintf(fid, '\t\t end \n');
fprintf(fid, '\t end \n');
fprintf(fid, ' end \n');

% T(i) contains the rate of infection received by node i. The following
% generates T(i) for each corner. If there are 2 graplets composed of a
% total of 10 corners then dim(T) = [1, 10].
T_count = 1;
for kk = 1:length(sg)
    if kk==3
        kk = 3;
    end
    state_card = length(sg{kk});
    for k = 1:state_card
        states = combinator(3,length(sg{kk}),'p','r')-2;
        line1 = 0;
        for i = 1:3^state_card
            if kk==1
                index_place = node_positions;
            else
                index_place = node_positions + sum(state_count(1:kk-1));
            end
            [no_inf] = inf_neighbors(k, states(i,:), sg{kk});
            if line1 == 0 && no_inf==1 && states(i,k)==-1
                fprintf(fid, 'T(%d) = tau*(y(%d)', [T_count i+index_place]);
                line1 = 1;
                T_count = T_count + 1;
            elseif line1 == 1 && no_inf==1 && states(i,k)==-1
                fprintf(fid, ' + y(%d)', i+index_place);
            elseif no_inf>0 && states(i,k)==-1
                fprintf(fid, ' +  %d*y(%d)', [no_inf i+index_place]);
            end
            if i== 3^state_card
                fprintf(fid,');\n');
            end
        end
    end
end

% The expected number of type-j hyperstubs infection will be
% exposed to upon infection of a node via any type of hyperstub is given by
% Delta(j), computed by the following:
fprintf(fid,'Delta = T*delta; \n \n');

% The following constructs a for' loop that defines the ODEs for the
% survivor functions (thetas):
fprintf(fid, ' for  j = 1:%d \n',node_positions);
fprintf(fid, '\t if PGF_Jacobian_1(j)==0 \n');
fprintf(fid, '\t\t dy(j) = 0; \n');
fprintf(fid, '\t else \n');
fprintf(fid, '\t\t dy(j) = -y(j)*T(j)/M(j); \n');
fprintf(fid, '\t end \n');
fprintf(fid, ' end \n \n');

% The following forms the ODEs for state transitions over subgraph
% types using the x_equations.m files. If the expected number of a subgraph
% type is zero it sets the corresponding ODEs to zero.
for i = 1:length(sg)
    if i==1
        index_place     = node_positions+1;
        index_place_i   = node_positions+1;
    else
        index_place   = index_place + length(sg{i-1});
        index_place_i   = index_place_i + 3^length(sg{i-1});
    end
    EQ_name    = sprintf('%s_equations',varargin{i});
    fprintf(fid, 'if PGF_Jacobian_1(%d) == 0 \n', index_place-node_positions);
    fprintf(fid, '\t dy(%d:%d) = 0; \n', [index_place_i index_place_i-1+3^(length(sg{i}))]);
    fprintf(fid, 'else \n');
    fprintf(fid, '\t [dy(%d:%d)] = %s',[index_place_i index_place_i-1+3^(length(sg{i})) EQ_name]);
    fprintf(fid, '(y(%d:%d)); \n',     [index_place_i index_place_i-1+3^(length(sg{i}))]);
    fprintf(fid, 'end \n \n');
end

% ODE for I:
fprintf(fid, 'dy(end-1) = -dot(dy(%d:%d),PGF_Jacobian_theta(%d:%d)) - gamma*y(end-1); \n', [1 node_positions 1 node_positions]);
% ODE for R:
fprintf(fid, 'dy(end) = gamma*y(end-1); \n');
fprintf(fid,'end');
fclose(fid);

% options = odeset('AbsTol',1e-8,'RelTol',1e-8);
% [T,Y] = ode45(@func,[0 Tend],alpha,options);
% % I will always be the 2nd to last varibale
% I = Y(:,end-1);
% % R will always be the last.
% R = Y(:,end);
% S = 1 - I - R;
```

```
end
```

## State transition matrix generation

```
function Z = trans_matrix(g,var_place,states)

% Generates the transition matrix. A matrix that contains the rates of
% transition from one subgraph configuration to another.
% INPUT: g, subgraph adjacency matrix
% OUTPUT: A cell array with the {i,j}th entry corresponding the probability
% that state(i) transitions to state(j).

nodes = length(g);
% -1 = S
%  0 = I
%  1 = R
card = length(states);
Z = cell(card);

for i = 1:card
    for k = 1:card
        if i~=k
            no_inf_events = 0;
            no_rec_events = 0;

            for j = 1:nodes;

                % If an S -> R, abort.
                if  states(i,j)==-1 && states(k,j)==1
                    Z{i,k} =0;
                    break
                end

                % If an I -> S, abort.
                if  states(i,j)==0 && states(k,j)==-1
                    Z{i,k} =0;
                    break
                end

                % If an R changes, abort.
                if states(i,j)==1 && states(k,j)~=1
                    Z{i,k} =0;
                    break
                end

                % if S -> I, find infectious pressure on S.
                if states(i,j) ==-1 &&  states(k,j)==0
                    no_inf_events = no_inf_events  +1;
                    no_inf = inf_neighbors(j, states(i,:), g);
                    if no_inf~=0
                        Z{i,k} = sprintf('%d*tau  + D(%d)/M(%d)',[no_inf j+var_place-1 j+var_place-1]);
                    else
                        Z{i,k} = sprintf('D(%d)/M(%d)',[j+var_place-1 j+var_place-1]);
                    end
                end

                % If I -> R, p(transition) = gamma
                if states(i,j) ==0 &&  states(k,j)==1
                    no_rec_events = no_rec_events + 1;
                    Z{i,k} = sprintf('gamma');
                end

                % If more than one even happens, p = 0.
                if no_inf_events > 1 || no_rec_events > 1 || (no_inf_events + no_rec_events)>1
                    Z{i,k} =0;
                    break
                end

            end
        end
    end
end
end
```

Published with MATLAB® R2013a
